# First-Line Bruton’s Tyrosine Kinase Inhibitor-Based Regimens for Mantle Cell Lymphoma

**DOI:** 10.3390/curroncol33070426

**Published:** 2026-07-17

**Authors:** Robert Puckrin, Diego Villa, Isabelle Fleury, Jean-François Larouche, John Kuruvilla

**Affiliations:** 1 Arthur J.E. Child Comprehensive Cancer Centre, University of Calgary, Calgary, AB T2N 5G2, Canada; 2 Centre for Lymphoid Cancer, BC Cancer, Vancouver, BC V5Z 1L3, Canada; 3 Division of Medical Oncology, Department of Medicine, University of British Columbia, Vancouver, BC V5Z 4E6, Canada; 4 Centre Hospitalier Universitaire de Québec, Université Laval, Québec City, QC G1J 5B3, Canada; 5 Division of Medical Oncology and Hematology, Princess Margaret Cancer Centre, Toronto, ON M5G 2M9, Canada

**Keywords:** mantle cell lymphoma, Bruton’s tyrosine kinase inhibitor, ibrutinib, acalabrutinib, zanubrutinib, chemotherapy-free, B-cell lymphoma 2

## Abstract

First-line (1L) therapy for mantle cell lymphoma (MCL) continues to evolve rapidly, with several recent phase II and phase III trials consistently showing the activity of novel covalent Bruton’s tyrosine kinase inhibitor (cBTKi) combinations. Historical treatment selection criteria such as patient age, fitness, and eligibility for autologous stem cell transplantation generally remain applicable for some, but not all, of these novel regimens. This potential for flexibility necessitates the consideration of additional factors during decision-making, such as MCL biology, patient risk tolerance, logistical and resource implications, and the impact on use of later-line options. This paper presents three illustrative patient cases to explore 1L therapy selection among these new and emerging cBTKi options. The realized and anticipated benefits, limitations, and challenges and persisting evidence gaps associated with these regimens are discussed.

## Simple Summary

Treatment of patients with mantle cell lymphoma is rapidly evolving, driven by several recent clinical trials showing that combining covalent Bruton’s tyrosine kinase inhibitors with other therapies can lead to improved outcomes. Although these newer treatment approaches expand available options and offer greater flexibility, choosing the best therapy has become more complex. Optimal treatment selection increasingly requires a personalized approach that considers disease features, patient health and preferences, and local factors such as access to care. In this paper, we present three patient cases to illustrate how these factors influence decision-making and to highlight the practical considerations involved in selecting among standard and emerging first-line covalent Bruton’s tyrosine kinase inhibitor-based combinations.

## 1. Background

Mantle cell lymphoma (MCL) is a rare, molecularly complex subtype of non-Hodgkin lymphoma that accounts for approximately 5% to 7% of lymphoma cases [1]. At diagnosis, median age ranges from 60 to 70 years, with most patients being male and presenting with advanced-stage disease [1,2]. Based on its heterogenous clinical course, MCL is categorized as either indolent or aggressive per classifications from the International Consensus Classification and the World Health Organization [3,4]. Regardless of classification, patients typically require multiple lines of therapy to manage their disease [5,6].

Historically, MCL has been associated with a poor prognosis, with a median overall survival (OS) of three to five years [2]. Over the past few decades, remarkable advances in the therapeutic landscape including the integration of dose-intensified chemoimmunotherapy (CIT) regimens, consolidative autologous stem cell transplantation (ASCT), and rituximab maintenance therapy have meaningfully improved clinical outcomes [5,7,8,9,10,11]. The introduction of covalent Bruton’s tyrosine kinase inhibitors (cBTKis, i.e., ibrutinib, acalabrutinib, and zanubrutinib), initially as monotherapy for relapsed/refractory (R/R) disease [12,13,14] and more recently as part of first-line (1L) combination regimens, has been similarly transformative. These targeted agents have undergone 1L evaluation in four recent phase III trials, including the TRIANGLE study of ibrutinib + R-CHOP/R-DHAP (rituximab–cyclophosphamide, doxorubicin hydrochloride, vincristine, prednisone/rituximab–dexamethasone, high-dose cytarabine, cisplatin) in younger, fit patients [15], as well as the SHINE (bendamustine + rituximab [BR] + ibrutinib) [16], ECHO (BR + acalabrutinib) [17], and ENRICH (ibrutinib + rituximab [IR]) [18] studies of older, unfit patients (Table 1). Additional cBTKi combinations, such as chemotherapy-free triplets including anti-B-cell lymphoma 2 (BCL-2) + cBTKi + anti-CD20 therapies, have undergone phase II evaluation (Table 1 and see Supplementary Table S1) [2,19,20,21].

Despite the advances afforded by novel 1L cBTKi regimens, MCL remains an incurable and often aggressive disease. Therapeutic responses continue to vary based on risk characteristics and toxicity remains a concern with intensive 1L regimens for many patients, leading to treatment avoidance, discontinuation, or non-relapse mortality (NRM) [22,23,24,25]. Furthermore, genomic instability and treatment resistance commonly increase over progressive lines of therapy, resulting in worsening disease kinetics and diminishing survival outcomes [6,19,26]. These considerations underscore the need to prioritize the best 1L therapies to provide long-lasting remission while also acknowledging potential implications for later-line options in terms of efficacy, toxicity, and drug access.

Recognizing these challenges, this paper synthesizes and compares recent practice-changing 1L clinical trials in MCL and examines the relative advantages and limitations of current and emerging regimens. A case-based discussion framework is utilized to discuss how these findings may be applied to real-world clinical practice in the setting of Canada’s publicly funded healthcare system.

**Table 1:** Selected phase III and phase II clinical trials evaluating 1L cBTKi combinations in MCL.

Study Name/Group NCT Number Author, Year	Intervention	Population *n* Median Age	Transplant Eligibility	Risk Characteristics ^a^				Median Follow-Up (mo)	ORR	CRR	PFS	OS
				MIPI High	Ki-67 ≥30%	*TP53*	Blastoid/ Pleomorphic Variants					
**Phase III Trials**												
TRIANGLE NCT02858258 Dreyling et al. 2024 [15] Dreyling et al. 2026 [27]	Arm A: R-CHOP/ R-DHAP (or R-DHAOx), followed by ASCT ± R maint Arm A + I: Per Arm A + I, ±IR maint Arm I: Per Arm A + I without ASCT ±IR maint	18–65 yrs *n* = 870 57 yrs	Eligible	Overall: 15% Arm A: 14% Arm A + I: 15% Arm I: 16%	Arm A: 33% Arm A + I: 31% Arm I: 32%	p53 >50%: Arm A: 12% Arm A + I: 14% Arm I: 15%	Blastoid: Arm A: 11% Arm A + I: 13% Arm I: 12%	54.9 (FFS)	Arm A + I + Arm I vs. Arm A: 98% vs. 94% (*p* = 0.0013)	Arm A + I + Arm I vs. Arm A: 44% vs. 36% (*p* = 0.037)	4-yr FFS (1-sided CI and *p*): A + I vs. I: 82% vs. 81% HR 0.86 (98.33% CI, 0.00–1.27; *p* = 0.21) A + I vs. A: 82% vs. 70% HR 0.63 (98.33% CI, 0.00–0.89; *p* = 0.0026) A vs. I: 70% vs. 81% HR 1.45 (98.33% CI, 0.00–2.02; *p* = 0.99)	4-yr OS: A + I vs. I: N/A A + I vs. A: 88% vs. 81% HR 0.59 (95% CI, 0.38–0.92; *p* = 0.0036) I vs. A: 90% vs. 81% HR 0.57 (95% CI, 0.36–0.90; *p* = 0.0019)
SHINE NCT01776840 Wang et al. 2022 [16]	I + BR vs. PBO + BR followed by R maint. (2 yrs) and I or PBO continued until PD/toxicity	Elderly (≥65 yrs) *n* = 523 71 yrs	Ineligible	s-MIPI: 35.6% vs. 33.2%	N/A	*TP53*m: 10% vs. 9.2%	7.3% vs. 9.9%	84.7	89.7% vs. 88.5%	65.5% vs. 57.6% (*p* = 0.06)	Median: 80.6 vs. 52.9 mo HR 0.75 (95% CI, 0.59–0.96, *p* = 0.01)	7-yr: 55.0% vs. 56.8% HR 1.07 (95% CI, 0.81–1.40)
ECHO NCT02972840 Wang et al. 2025 [17] Dreyling et al. 2024 (Abs) [28] Wang et al. 2025 (Abs) [29]	A + BR vs. PBO + BR followed by R maint. (2 yrs) and I or PBO continued until PD/toxicity	Elderly (≥65 yrs) *n* = 598 71 yrs	Ineligible	s-MIPI: 24.1% vs. 24.4%	46.5% vs. 49.2%	*TP53*m: 7.4% vs. 9.7%	13.7% vs. 12.7%	60.8 (reverse KM method for PFS)	91.0% vs. 88.0%	66.6% vs. 53.5%	Median: 72.5 vs. 47.8 mo Stratified HR 0.68 (95% CI, 0.53–0.87; *p* = 0.002)	Median: NR 36-mo: 73.8% vs. 68.3% Stratified HR 0.87 (95% CI, 0.67–1.13)
ENRICH Phase II/III NCT11038174 Lewis et al. 2025 [18]	IR vs. R-CHOP or BR (clinician choice)	Elderly (≥60 yrs) *n* = 397 74 yrs	Ineligible	56% (222/393)	42% (126/299)	*TP53*m: 26% (40/155)	Blastoid: 7% (25/370)	47.9	86% vs. 85%	54% vs. 53%	Median: 65.3 vs. 42.4 mo HR 0.69 (95% CI, 0.52–0.90; *p* = 0.003) ^b^	5-yr: 58% vs. 55% HR 0.87 (95% CI, 0.64–1.18)
**Phase II Trials**												
RECTANGLE NCT04566887 Villa et al. 2023 (Abs) [30]	AR-CHOP, then ASCT and maint. AR (2 yrs) in responders	Fit, ≥18 yrs *n* = 54 (results for 43 assessed pts) 62 yrs	Eligible	14%	26%	N/A	16%	11.8	100%	91%	12-mo: 94%	12-mo: 95.5%
ALTAMIRA (NLG-MCL8) NCT05214183 Jerkeman et al. 2024 (Abs + Presentation) [31]	AR vs. matched external cohort (MCLcomplete)	Elderly (≥60 yrs) *n* = 81 75 yrs	Ineligible	69%	>30%: 22%	24%	11%	15.5	6-mo: 76%	6-mo: 51%	1-yr: 90% 2-yr: 75% (improved vs. control arm) 2-yr by risk High risk: 32% Low risk: 91%	1-yr: 94% 2-yr: 84% (improved vs. control arm)
BOVen NCT03824483 Kumar et al. 2025 [32]	Z + O + V; treatment D/C based on uMRD status	≥18 yrs, *TP53*m *n* = 25 68 yrs	Not specified	68%	52%	100%	Blastoid: 20%	28.2	96% (best)	88%	2-yr: 72% Disease-specific 2-yr: 91%	2-yr: 76%
TrAVeRse NCT05951959 Hawkes et al. 2025 (Abs) [33]	AVR; pts with MRD-CR randomized to A or observation until PD	≥18 yrs *n* = 108 69 yrs	Not specified	s-MIPI: 30.6%	38.0%	*TP53*m: 19.1% (17/89)	8.3%	10.5	95.4% (103/108) *TP53*m: 88.2% (15/17)	53.7% (58/108; Lugano) *TP53*m: 64.7% (11/17)	6-mo: 97.1%	6-mo: 98.1%
SYMPATICO NCT03112174 Wang et al. 2025 (Abs) [34]	I + V for 2 yrs, then I until PD/toxicity	≥65 yrs or ≥18 yrs and *TP53*m *n* = 78 83% ≥65 yrs	Not specified	s-MIPI: 45%	N/A	*TP53*m: 37% (*n* = 29)	N/A	40.5 (median time on study)	95% *TP53*m: 90%	69% *TP53*m: 55%	Median: 40.2 mo *TP53*m: 22 mo	3-yr: 79% *TP53*m: 68%

Information presented from most recent source(s)/data cuts available at the time of publication. Cross-trial comparisons should be conducted with caution given differences in study designs, patient populations, interventions, endpoint definitions, and methods of analysis. ^a^ Denominators vary for some parameters. ^b^ Significant interaction observed between choice of CTX and treatment effect for PFS: IR superior to R-CHOP (HR 0.37; 95% CI, 0.22–0.62) but comparable to BR (HR 0.91; 95% CI, 0.66–1.25). 1L, first line; A, acalabrutinib; Abs, abstract; ASCT, autologous stem cell transplantation; BR, bendamustine + rituximab; cBTKi, covalent Bruton’s tyrosine kinase inhibitor; CI, confidence interval; CR(R), complete response (rate); CTX, chemotherapy; D/C, discontinuation; FFS, failure-free survival; HR, hazard ratio; I, ibrutinib; KM, Kaplan–Meier; maint., maintenance; MCL, mantle cell lymphoma; (s/b/c)-MIPI, simplified, biologic, or combined MCL International Prognostic Index; mo, month(s); MRD, minimal residual disease; N/A, not available; NR, not reached; O, obinutuzumab; ORR, objective/overall response rate: OS, overall survival; PBO, placebo; PD, progressive disease; PFS, progression-free survival; pts, patients; R, rituximab; R-CHOP, rituximab–cyclophosphamide, doxorubicin hydrochloride, vincristine, prednisone; R-DHAOx, rituximab–dexamethasone, high-dose cytarabine, and oxaliplatin; R-DHAP, rituximab–dexamethasone, high-dose cytarabine, cisplatin; *TP53*m, *TP53* mutation-positive; V, venetoclax; yr(s), year(s); Z, zanubrutinib.

## 2. Case-Based Discussions

### 2.1. Overview

The considerations and treatment choices presented herein reflect the personal opinions of the co-authors, which have been informed by the totality of evidence from phase II and III clinical trials in 1L MCL (and in some cases, other hematological cancers) and experience derived from real-world clinical practice. The co-authors acknowledge the significant limitations associated with cross-trial comparisons and the need to optimize 1L treatment while minimizing toxicity whenever possible. They also recognize the importance of shared decision-making (SDM) with patients and emphasize that individual patient priorities related to treatment duration (e.g., convenience, toxicity risk, cost), efficacy, and tolerability must be factored into the selection of 1L and subsequent-line therapies.

### 2.2. Illustrative Case 1: Classic MCL (Table 2)

At 65 years of age, this patient lies near the eligibility threshold for the TRIANGLE, SHINE, and ECHO clinical trials. The potential benefits and risks of each approach should therefore be carefully weighed via individualized treatment decision-making.

#### 2.2.1. Treatment Selection Considerations: I + R-CHOP/R-DHAP Then Maintenance IR (TRIANGLE)

TRIANGLE was a large (*n* = 870), randomized study in which ibrutinib–CIT combination therapy showed favorable efficacy outcomes, including a high overall response rate (ORR) and failure-free survival (FFS) and OS benefits at a median follow-up of 54.9 months (Table 1) [15,27]. Given the demonstration of a statistically significant OS benefit, its results are viewed as potentially more robust than those from SHINE and ECHO [16,17], although differences between trial populations in terms of age and fitness are acknowledged. Patient fitness is an especially critical determinant of treatment selection, since R-CHOP/R-DHAP is a more intensive regimen than BR and is associated with greater toxicity including myelosuppression, infection, renal injury, cardiotoxicity, hospitalization, and transfusion requirements [15,36]. If neuropathy is not a concern, R-DHAOx (rituximab–dexamethasone, high-dose cytarabine, and oxaliplatin) could be used instead of R-DHAP given the favorable safety profile of oxaliplatin compared with cisplatin and the inclusion of both regimens in TRIANGLE [37,38,39]. Additional support for R-DHAOx is provided by a post hoc analysis of the LyMA trial in 1L MCL, which showed oxaliplatin to be associated with significantly improved PFS and OS versus cisplatin and an independent favorable prognostic factor alongside a low MCL International Prognostic Index (MIPI) score [37].

An additional factor supporting selection of the TRIANGLE regimen is its fixed duration of administration. Compared with continuous use of cBTKi therapy in both SHINE and ECHO, two years of maintenance ibrutinib in TRIANGLE is likely to limit cumulative toxicity, treatment resistance, healthcare resource utilization (HCRU), and costs, as observed with fixed-duration approaches in chronic lymphocytic leukemia (CLL) [40,41,42,43]. This fixed-duration approach may also have increased appeal to patients, physicians, and payers [44,45], and theoretically preserves the option of cBTKi re-treatment at relapse, assuming an absence of disease progression while therapy was actively being received. However, data for cBTKi re-challenge outcomes among patients who relapse post-TRIANGLE are currently unavailable, including those demonstrating the optimal timing of re-treatment and the impact of previous cBTKi exposure on subsequent resistance. Potential benefits notwithstanding, approximately 2.5 years of cBTKi exposure may still be associated with cumulative toxicity (e.g., infection) and can have implications for vaccine response, as shown in CLL [40,46,47], underscoring the importance of monitoring and supportive care to mitigate adverse events (AEs).

In contrast to the known lymphodepleting effects of BR discussed below, selection of TRIANGLE may also preserve T-cell fitness and eligibility for 2L chimeric antigen receptor (CAR)-T cell therapy for patients who progress on cBTKi therapy. Although CAR-T cell therapy is approved for third-line (3L) use in Canada [48], patients with only one prior line of therapy were enrolled in the ZUMA-2 trial of brexucabtagene autoleucel [49]; accordingly, regional CAR-T cell funding criteria might support 2L administration for the rare patient who progresses during the TRIANGLE protocol.

Importantly, the TRIANGLE regimen incorporates ibrutinib, a first-generation cBTKi associated with poorer tolerability than second-generation cBTKis. Extrapolation of findings from the phase III ELEVATE-RR, ALPINE, and ASPEN trials in CLL and Waldenström’s macroglobulinemia suggests that ibrutinib may have a higher risk of cardiovascular (CV) events, other severe AEs, and treatment discontinuation for toxicity compared with both acalabrutinib and zanubrutinib [50,51,52,53,54]. Accordingly, patients’ CV risk profile should be considered before selecting TRIANGLE combination therapy and substitution of ibrutinib for a second-generation cBTKi may be considered, although no data are available to support this approach. First-line evaluation of acalabrutinib + R-CHOP in MCL is currently underway in the Canadian RECTANGLE trial (Table 1) [30].

Although this patient may have previously been considered for high-dose chemotherapy (HDT) followed by ASCT, in TRIANGLE, patients who received I + R-CHOP/R-DHAP without HDT-ASCT achieved comparable complete response (CR), FFS, and OS rates to those who received I + R-CHOP/R-DHAP + HDT-ASCT [15,27] (Table 1). These patients also experienced fewer HDT-ASCT-related toxicities such as cytopenias and infection. The opportunity to avoid HDT-ASCT-related toxicity while preserving efficacy makes the A + I arm of TRIANGLE an appealing option for this 65-year-old individual.

#### 2.2.2. Treatment Selection Considerations: BR + cBTKi (SHINE, ECHO)

The BR + cBTKi combinations used in SHINE and ECHO are appropriate for patients who are ineligible for intensive CIT or ASCT, as they can prolong PFS and potentially offer an improved tolerability profile compared with the TRIANGLE regimen. The BR backbone is associated with more favorable safety data than R-CHOP/R-DHAP and is therefore preferable for older and/or comorbid patients. Furthermore, selection of BR + cBTKi therapy provides an opportunity to access a second-generation cBTKi (acalabrutinib in ECHO [17]) that is not otherwise publicly funded for MCL in Canada at this time [55]. The fully outpatient mode of administration of the BR + cBTKi regimens may also be appealing, whereas use of cytarabine per TRIANGLE may require inpatient therapy depending on local resources. Similarly, use of BR can avoid the need for indwelling central venous access devices, which may be necessary for administration of high-dose cytarabine in some patients [56,57].

Despite favorable safety and PFS results, an OS benefit has not been shown in SHINE or ECHO. A trend towards inferior OS was observed with BR + ibrutinib in SHINE at seven years, while a numerically favorable OS trend was reported for BR + acalabrutinib in ECHO at three years, a similar timepoint to TRIANGLE (Table 1) [16,17]. Nevertheless, PFS is clinically meaningful for both patients and physicians and is a benchmark upon which many other MCL therapies have been established.

In both SHINE and ECHO, cBTKi therapy was provided continuously until progressive disease (PD) or intolerance, which has implications for cumulative toxicity, resistance, and costs [40]. Use of BR + continuous cBTKi combinations may also limit salvage options for patients who experience PD while on treatment, as cBTKi resistance may result in ineligibility for ibrutinib + venetoclax (I + V) combinations. This issue may be circumvented in the future, however, with the emergence of novel alternatives such as CAR-T cell therapy, non-covalent BTKi options (pirtobrutinib) [58], and bispecific antibodies (e.g., glofitamab and odronextamab [59,60]).

Finally, if this patient had presented with high-biologic-risk MCL, bendamustine may be a suboptimal partner to cBTKi therapy. This is because of its reduced efficacy in aggressive subtypes [61,62] and concern that its prolonged lymphodepleting effects could impair immune reconstitution and compromise subsequent CAR-T cell manufacturing and efficacy [63,64,65].

### 2.3. Illustrative Case 2: Standard-Risk MCL (Two Scenarios) (Table 3 and Table 4)

Given this patient’s older age, he would be considered ineligible for the TRIANGLE protocol at most centers but could be a candidate for either 1L BR followed by cBTKi at disease relapse or the 1L combination of BR + cBTKi therapy.

#### 2.3.1. Treatment Selection Considerations: BR (With cBTKi Reserved for Relapse; StiL-1, BRIGHT, SHINE/ECHO Control Arms)

The use of BR with or without maintenance rituximab is associated with fewer toxicities and a shorter duration of active combination therapy than BR + cBTKi, without a detriment in OS [16,17,66,67]. However, PFS is inferior with BR alone: patients who do not receive concomitant cBTKi therapy are more likely to relapse earlier and require subsequent 2L and 3L therapies [16,17,29]. Nevertheless, the median improvements in PFS1 observed in the SHINE and ECHO trials (approximately 25 to 28 months) are comparable to the median PFS2 achieved with single-agent BTKi therapy in the relapsed setting, highlighting the feasibility of a sequential strategy in which patients treated with BR alone can be effectively salvaged with a cBTKi at relapse. Additionally, the absence of an OS benefit in SHINE and ECHO suggests that some patients with MCL derive similar long-term outcomes with 1L BR followed by cBTKi at relapse, supporting the viability of both treatment strategies [16,17,66,67,68].

Reserving cBTKi therapy for the 2L setting may also allow for a break from continuous treatment after 1L BR, recognizing that many patients remain on maintenance rituximab during this period. Such breaks may help reduce cumulative toxicities and overall healthcare costs and improve patient health-related quality of life (HRQoL) [12,69,70]. Additionally, patients and physicians may feel reassured that a proven 2L cBTKi option or more effective novel 2L combinations (e.g., I + V) will be available at the time of relapse. However, some patients do not respond to 2L cBTKi therapy, presenting a challenging clinical scenario, and a proportion of patients, particularly those with very aggressive disease, may never proceed to 2L options [71,72,73]. As such, upfront incorporation of cBTKi therapy ensures reception of this effective drug class and may help maximize 1L disease control while delaying first and second relapse.

#### 2.3.2. Treatment Selection Considerations: 1L BR + cBTKi Combinations (SHINE, ECHO)

Compared with 1L use of BR + maintenance rituximab alone, the BR + cBTKi + maintenance rituximab combination regimens have a key advantage of prolonging PFS by a median of about two years (Table 1) [16,17]. Furthermore, in a recent analysis of the ECHO trial, incorporation of acalabrutinib into 1L therapy reduced not only the risk of requiring 2L (11% vs. 33%) but also 3L therapy (3% vs. 12%) compared with use of BR followed by BTKi at relapse [29]. The median time to 3L therapy or death was not reached in the acalabrutinib arm versus 73.8 months in the placebo arm (stratified HR 0.76; 95% CI 0.59–0.98), suggesting that optimization of 1L treatment may provide long-term disease control. Despite these benefits, indefinite 1L cBTK inhibition until disease progression or unacceptable intolerance per SHINE or ECHO may increase the risk of cumulative toxicities, premature treatment discontinuation, treatment resistance, and/or costs, and may not be preferred by some patients. Nonetheless, these concerns may be partially offset by access to acalabrutinib via ECHO, given that the safety profile of BR + acalabrutinib appears to be preferable to that of BR + ibrutinib in SHINE.

Considering the absence of an OS difference between these 1L BR and BR + cBTKi strategies and their associated trade-offs, this case underscores the importance of SDM with patients who are eligible for either approach. Careful consideration should be given to balancing patient characteristics, disease risk, treatment duration, efficacy, tolerability, and access. Some patients may prefer a treatment break and reserving cBTKi for relapse, while others may prioritize disease control by receiving all three drug classes upfront.

**Table 4:** Case 2: Key clinical features and treatment options under consideration for a patient with standard risk MCL—Scenario B.

SCENARIO B: Treatment Options Under Consideration			
BR + cBTKi Combination (SHINE and ECHO Trials)		Chemotherapy-Free Strategy Ibrutinib + Rituximab (ENRICH Trial)	
Key Advantages	Key Disadvantages	Key Advantages	Key Disadvantages
Superior efficacy compared with BR alone Suitable for patients without major comorbidities Opportunity to access second-generation cBTKi (ECHO)	Indefinite cBTKi therapy increases safety risks, costs, and treatment resistance Lack of OS benefit Potential toxicity, especially with ibrutinib	Efficacy is comparable to that of BR Avoids chemotherapy and related toxicity Fast HRQoL improvement	Indefinite cBTKi therapy increases safety risks, costs, and treatment resistance Lack of OS benefit Lack of PFS benefit vs. SoC BR CV toxicity of first-generation cBTKi ibrutinib

BR, bendamustine + rituximab; cBTKi, covalent Bruton’s tyrosine kinase inhibitor; CV, cardiovascular; HRQoL, health-related quality of life; OS, overall survival; PFS, progression-free survival; SoC, standard of care.

The recently established strategy of BR + cBTKi therapy with maintenance rituximab has been challenged by the chemotherapy-free IR regimen evaluated in the ENRICH trial. This combination offers an additional 1L option for this patient case and makes the optimal approach for older and/or less fit MCL populations uncertain.

#### 2.3.3. Treatment Selection Considerations: BR + cBTKi Combination (SHINE, ECHO)

Although direct comparative data are currently unavailable, the BR + cBTKi combination may be more effective than IR as it was associated with prolonged PFS compared with BR in both SHINE and ECHO [16,17]; in contrast, PFS with IR was only comparable to that with BR in the ENRICH trial (Table 1) [18]. Additionally, distinct safety profiles are associated with the two combinations. Increased neutropenia may be observed with BR; however, this AE is more predictable and more easily managed than potentially serious ibrutinib-related CV complications. Importantly, selection of the ECHO protocol permits access to 1L acalabrutinib, which again may be better tolerated than ibrutinib and is only approved for use in combination with BR and not as monotherapy [74].

#### 2.3.4. Treatment Selection Considerations: Ibrutinib + Rituximab (ENRICH)

ENRICH is the first randomized, phase II/III trial to evaluate a 1L chemotherapy-free strategy in MCL [18], with previous evidence for this approach being limited to earlier-phase studies (Table 1 and Table S1) [18,31,33,75,76,77]. In ENRICH, IR was associated with superior PFS compared with CIT, although the benefit appeared to be restricted to the R-CHOP subgroup of the control arm; as noted previously, no significant PFS benefit was observed versus BR [18].

Chemotherapy-free strategies may be of interest for high biologic-risk subsets who respond poorly to cytotoxic therapy, such as patients with a *TP53* mutation (*TP53*m). However, exploratory post hoc analyses from ENRICH show that the benefit of ibrutinib may paradoxically be greater among low-risk patients (e.g., Ki-67 < 30%), while blastoid variants experience improved PFS with CIT, although analyses were limited by a small sample size [18]. Additionally, while the IR combination may be preferred by patients who wish to avoid chemotherapy-related toxicities, the regimen is not free of risk. For example, although grade ≥3 hematological toxicity was considerably reduced with IR, overall grade ≥3 AE rates were higher, a finding that may partially reflect the longer treatment exposure associated with continuous ibrutinib administration until disease progression or unacceptable toxicity [18]. The trial also showed that CV toxicity remained a particular concern with ibrutinib, with higher rates of atrial fibrillation observed with IR than with CIT (grade ≥3 AF: 7% vs. 1%, respectively). Despite these findings, patient HRQoL improved faster with IR and was higher than with CIT at mid-treatment, although long-term HRQoL outcomes were similar between groups [18,19]. Extended follow-up is required to determine whether ibrutinib is associated with a lower risk of secondary malignancies, a key concern with CIT [78]. Overall, second-generation cBTKi–rituximab regimens are anticipated to offer a better-tolerated approach than IR, including the combinations of acalabrutinib + rituximab being evaluated in the phase II ALTAMIRA [31] and CARAMEL (NCT05004064) trials and zanubrutinib + rituximab in the phase III MANGROVE trial [79]. In ALTAMIRA, patients with low-risk biology had a substantially greater PFS benefit with acalabrutinib + rituximab than those with high-risk MCL (Table 1), a finding comparable to the results of ENRICH [18,31].

As this patient is suitable for either the BR + cBTKi or the IR regimen, neither option has a proven OS benefit, and each involves continuous use of cBTKi therapy, the choice of regimen will require balancing trade-offs in the context of an individual patient’s circumstances. For a patient who is frailer than the current case, the chemotherapy-free regimen may be preferable because BR may be poorly tolerated; for a fitter patient, BR + cBTKi may be preferable given the data for PFS.

### 2.4. Illustrative Case 3: High-Risk MCL (Table 5)

#### 2.4.1. Characteristics of High-Risk MCL

The identification of high-risk MCL is determined through the assessment of clinical and molecular prognostic factors [22,23]. For patients with newly diagnosed disease, the most important high-risk characteristics include the presence of *TP53*m (confirmed by next-generation sequencing [NGS]), high Ki-67 (≥30–50%), high MCL International Prognostic Index (MIPI) score (≥6.2) [35], and aggressive histology (blastoid or pleomorphic) [22,23]. Additional high-risk characteristics include complex karyotype, central nervous system involvement with systemic disease, bulky disease, del(17p) by fluorescence in situ hybridization test, p53 protein overexpression by immunohistochemistry (IHC), and the presence of other genetic mutations such as those in *CCND1*, *CDKN2A*, *NOTCH1/2*, *KMT2D*, *NSD2*, and *SMARCA4* [22]. Patients with these prognostic factors, especially *TP53*m-positive disease, have the greatest need for new treatment options [22,23,24].

**Table 5:** Case 3: Key clinical features and treatment options under consideration for a patient with high-risk MCL.

**Key Clinical Features:** A 58-year-old female Diagnosis: Previously untreated stage IV MCL ECOG PS: 1 MIPI: 6.3 (high risk) ^a^ Morphology: Pleomorphic Ki-67: 60% *TP53*m status: positive (confirmed by NGS) Past medical history: No relevant comorbidities			
**Treatment Options Under Consideration**			
**I + R-CHOP/R-DHAP ^b^ ± ASCT then Maintenance IR** **(TRIANGLE trial)**		**cBTKi-BCL2i Combinations** **(e.g., BOVen, TrAVeRse, SYMPATICO trials)**	
**Key Advantages**	**Key Disadvantages**	**Key Advantages**	**Key Disadvantages**
Robust evidence from phase III randomized trial FFS and OS benefit Fixed-duration regimen Opportunity for cBTKi re-treatment at relapse	*TP53*m status was evaluated using IHC, not NGS Patients with p53 protein overexpression represented a small proportion of overall trial population CIT component is known to have limited benefit in *TP53*m-positive disease Regimen includes first-generation cBTKi associated with increased toxicity	Best-available evidence for *TP53*m-positive MCL, showing favorable response rates, uMRD, PFS, and/or safety profiles Avoids use of cytotoxic agents	Lack of 1L, phase III, randomized clinical trials Small population sizes in BOVen and TrAVeRse Some regimens require indefinite use of cBTKi therapy ^c^ Regimens yet to receive regulatory approval (worldwide) Limited clinical trial follow-up time

^a^ Cut-offs based on Hoster et al. (2008) [35]; ^b^ R-DHAP could be substituted for R-DHAOx; ^c^ BOVen permitted treatment discontinuation based on achievement of uMRD, while in TrAVeRse, patients with a CR and uMRD were randomized to discontinuation or ongoing acalabrutinib. 1L, first-line; ASCT, autologous stem cell transplant; BCL2i, B-cell lymphoma 2 inhibitor; cBTKi, covalent Bruton’s tyrosine kinase inhibitor; CIT, chemoimmunotherapy; CR, complete response; ECOG, Eastern Cooperative Oncology Group; FFS, failure-free survival; I, ibrutinib; IHC, immunohistochemistry; MCL, mantle cell lymphoma; MIPI, MCL International Prognostic Index; NGS, next-generation sequencing; OS, overall survival; PFS, progression-free survival; PS, performance status; R, rituximab; R-CHOP, rituximab–cyclophosphamide, doxorubicin hydrochloride, vincristine, and prednisone; R-DHAOx, rituximab–dexamethasone, high-dose cytarabine, and oxaliplatin; R-DHAP, rituximab–dexamethasone, high-dose cytarabine, and cisplatin; *TP53*m, *TP53* mutation; uMRD, undetectable minimal residual disease.

This younger and fit patient with high-risk, *TP53*m-positive MCL is unlikely to achieve durable disease control with traditional CIT approaches [24,80]. The emergence of regimens combining cBTKi therapy with CIT (TRIANGLE) or BCL2 inhibition (e.g., BOVen, TrAVeRse) offers alternative 1L options, each with distinct advantages and limitations. At present, many countries lack access to chemotherapy-free doublet and triplet regimens, although this situation may evolve with the emergence of additional data.

#### 2.4.2. Treatment Selection Considerations: I + R-CHOP/R-DHAP ± ASCT Then Maintenance IR (TRIANGLE)

Patients with high-risk MCL may benefit from upfront use of multi-agent regimens that incorporate therapies with complementary mechanisms of action. Accordingly, integration of a cBTKi into 1L therapy, as done in the TRIANGLE trial, may be attractive for individuals who are expected to derive limited benefit from CIT alone. In TRIANGLE, subgroup analyses of patients with high p53 expression (>50%) and other adverse-risk characteristics suggested that FFS was improved among those who received ibrutinib compared with those who did not [15,27]. However, the study was not stratified to formally assess efficacy based on high-risk features, including *TP53*m-positive disease, and the subgroup analyses were limited by small sample sizes (e.g., because high-risk biologic disease features were not assessed in all patients) and reduced statistical power. Additionally, p53 status was assessed by IHC rather than NGS, which can result in misclassification of risk. Although use of ibrutinib appears to improve survival outcomes, it remains unclear whether the toxicities associated with the intensive R-CHOP/R-DHAP backbone used in TRIANGLE are justified in *TP53*m-positive MCL, where intensified chemotherapy has not been associated with sustained disease control [24].

If the TRIANGLE regimen is selected for this patient case, several factors should be considered to inform inclusion of ASCT after induction therapy. Firstly, although long-term data for CIT followed by ASCT with or without subsequent rituximab maintenance have shown sustained remission and potential for functional cure in a significant proportion of patients with MCL [7,81,82], ASCT can cause considerable toxicity, requires substantive resource and social support, may compromise the fitness of T cells for subsequent CAR-T cell therapy, and typically provides limited efficacy in individuals with *TP53*m-positive disease [7,83,84,85]. Additionally, in the overall trial population of TRIANGLE, no FFS or OS detriment was associated with omitting ASCT; however, subgroup analyses suggested that ASCT plus ibrutinib (the “A + I” arm) was potentially associated with improved FFS compared with ibrutinib (the “I” arm) in higher-risk patient subsets, including those with high Ki-67, blastoid or pleomorphic histology, and/or high p53 expression [15,27,86], although these post hoc analyses were underpowered. Overall, the optimal role of ASCT consolidation remains unclear for patients with high-risk *TP53*m-positive disease [83]. Although the current follow-up duration of TRIANGLE is limited to approximately four years and thus the long-term durability of CIT + cBTKi without ASCT remains uncertain, given similar early efficacy outcomes and toxicity considerations, it may be reasonable to omit ASCT for most patients with MCL who receive TRIANGLE induction with 1L cBTKi therapy. ASCT can also be omitted for patients who achieve MRD-negative status after induction, as shown in the EA5141 trial, which used the ClonoSeq assay [87]. The inclusion of ASCT in the TRIANGLE protocol should therefore be discussed with high-risk patients on a case-by-case basis and only considered for those who accept the increased toxicity risk [83,84].

Another consolidation option, allogeneic stem cell transplantation (alloSCT), is generally considered inappropriate in the 1L for most patients with MCL given an elevated risk of NRM, graft-versus-host disease (GVHD), and infection, among other concerns. Still, given the dismal prognosis of *TP53*m-positive MCL and the lack of availability of CAR-T cell therapy until progression, fit and motivated patients who achieve a response to 1L therapy may be considered for consolidation alloSCT on an individual basis, as it remains one of the only therapies to demonstrate curative potential in MCL, including in a small retrospective series of *TP53*m-positive cases [88]. Such patients must be accepting of the risks related to NRM, GVHD, and infection.

#### 2.4.3. Treatment Selection Considerations: cBTKi-BCL2i Combinations (BOVen, TrAVeRse, SYMPATICO)

As patients with *TP53*m-positive MCL may experience poor outcomes with CIT ± cBTKi therapy, novel targeted regimens are increasingly preferred and anticipated to have a more favorable safety profile than intensive combination chemotherapy. Individuals with high-risk MCL frequently show overexpression of BCL2 that contributes to apoptosis resistance, especially patients with blastoid-variant, *TP53*m-positive disease [89]. The cBTKi-BCL2i combination therapy is recognized to enhance apoptosis and induce deeper remissions through complementary targeting of survival pathways [90,91].

Although multiple phase II trials have recently shown promising efficacy with cBTKi-BCL2i combinations in MCL, the inclusion of patients with high-risk characteristics varies considerably (Table 1 and Table S1) [32,33,34,75,76,77,92,93]. Strict eligibility criteria and the time required for trial screening and enrollment have likely resulted in underrepresentation of patients with the most rapidly progressing or high-risk presentations of the disease. The phase II BOVen trial of zanubrutinib, venetoclax, and obinutuzumab combination therapy represents the only prospective study specifically dedicated to newly diagnosed, NGS-defined *TP53*m-positive MCL (Table 1) [92]. The trial’s two-year PFS rate of 72% compares favorably with outcomes observed in other *TP53*m-positive MCL cohorts [24], and MRD negativity was high at initial follow-up (28.2 months, cycle 13: 95% of patients at 1 × 10^−5^ sensitivity; 84% at 1 × 10^−6^). The trial’s inclusion of treatment guidance based on undetectable MRD status is of interest, with results suggesting that even high-risk patients may have an opportunity to stop therapy. Still, data are limited by the relatively small study population (*n* = 25) and the absence of a comparator group. Another study, the phase II TrAVeRse trial (N = 108), offers further evidence for the efficacy and safety of a second-generation cBTKi-BCL2i combination, showing favorable response and safety outcomes with 1L acalabrutinib, venetoclax, and rituximab therapy in both the overall population and among patients with *TP53*m-positive disease (*n* = 17/89 tested) (Table 1) [33]. Additionally, combination I + V therapy was validated for 1L treatment of *TP53*m-positive MCL in the phase II SYMPATICO trial, which showed an ORR of 90%, a CR rate of 55%, and a median PFS of 22 months in a 29-patient cohort [34]. A PFS benefit was also observed with I + V versus ibrutinib alone among patients with *TP53*m-positive R/R MCL in the trial’s randomized cohorts [91].

## 3. Conclusions

Evidence supporting the use of novel cBTKi-based combinations, including both CIT-paired and chemotherapy-free approaches, is rapidly expanding and may further improve 1L outcomes for patients with MCL. Across multiple phase II and phase III trials, these regimens have been associated with clinically meaningful benefits, including improved outcomes in patient groups for whom options have historically been limited, such as older, frail, and ASCT-ineligible populations and those with high-risk, *TP53*m-positive disease.

As illustrated by the cases discussed herein, several areas of uncertainty remain and warrant further investigation. These include a lack of direct comparative evaluation between some treatment protocols, the relatively short trial follow-up durations of current studies, limited data for certain patient subsets (particularly those with high-risk disease biology), and uncertainty regarding the optimal sequencing of cBTKis in MCL. In the absence of long-term and/or head-to-head comparative evidence for contemporary strategies such as TRIANGLE, ECHO, and ENRICH, treatment selection should be individualized and guided by SDM that incorporates a multitude of considerations. These may include patient age, fitness, and comorbidities, disease-related risk characteristics, treatment duration and toxicity profile, convenience (e.g., family, caregiver, travel, and work impact), logistical feasibility and resource requirements, regulatory approval and reimbursement criteria, and impact on subsequent therapeutic options (including CAR-T cell therapy eligibility), as well as patient preferences and goals of care. Patients should be counseled on relevant treatment options and their anticipated benefits and risks over both the short and long term. Analyses of patient perceptions of short-term toxicity versus potential for longer-term survival benefits are of interest in this setting.

In Canada, delays in obtaining drug funding and interprovincial and regional differences in infrastructure and geography can influence real-world access and feasibility, especially for therapies that require inpatient administration or intensive monitoring. These factors can affect not only reimbursement, but also treatment convenience and the practical burden of care for patients in rural or remote settings. Ongoing evaluation of cBTKi combinations through clinical trials and real-world data collection and real-world evidence generation is expected to clarify long-term outcomes, including efficacy, safety, HRQoL, and HCRU, and better support access. Such data may also further inform the value of combinations that incorporate second-generation cBTKis and other emerging agents, outcomes in *TP53*m-positive disease and other high-risk indicators, the potentially revised role of ASCT, selection of chemotherapy-free regimens, integration of fixed-duration approaches or other stopping rules (e.g., upon achievement of MRD negativity), and optimized drug sequencing with consideration of current and expected later-line options. Collectively, ongoing and emerging data will further refine the evidence base and support increasingly personalized 1L treatment selection for patients with MCL.

## Figures and Tables

**Table 2:** Case 1: Key clinical features and treatment options under consideration for a patient with classic MCL.

**Key Clinical Features:** A 65-year-old male Diagnosis: Previously untreated stage IV MCL with symptomatic lymphadenopathy ECOG PS: 1 MIPI: 6.0 (intermediate risk) ^a^ Morphology: Classic Ki-67: 40% *TP53*m status: Wild type Past medical history: Well-controlled hypertension, dyslipidemia, and diabetes mellitus			
**Treatment Options Under Consideration**			
**I + R-CHOP/R-DHAP ^b^, then Maintenance IR** **(TRIANGLE trial)**		**BR + cBTKi Combination** **(SHINE and ECHO trials)**	
**Key Advantages**	**Key Disadvantages**	**Key Advantages**	**Key Disadvantages**
Large trial with favorable efficacy outcomes, including OS benefit Fixed-duration regimen Opportunity for cBTKi re-treatment at relapse Opportunity to avoid ASCT without compromising efficacy	R-CHOP/R-DHAP associated with higher toxicity and HCRU than BR Inclusion of first-generation cBTKi associated with increased toxicity	Bendamustine is generally well tolerated and avoids toxicity and HCRU burden associated with intensive chemotherapy Opportunity to access second-generation cBTKi acalabrutinib with improved safety profile (ECHO)	Potential toxicity, especially with ibrutinib Lack of OS benefit Indefinite cBTKi therapy increases safety risks, costs, and treatment resistance Limited treatment options upon disease progression Bendamustine exposure may impact CAR-T cell therapy manufacturing

^a^ Cut-offs based on Hoster et al. (2008) [35]; ^b^ R-DHAP could be substituted for R-DHAOx. ASCT, autologous stem cell transplantation; BR, bendamustine + rituximab; cBTKi, covalent Bruton’s tyrosine kinase inhibitor; CAR, chimeric antigen receptor; ECOG PS, Eastern Cooperative Oncology Group performance status; HCRU, healthcare resource utilization; I, ibrutinib; MCL, mantle cell lymphoma; MIPI, MCL International Prognostic Index; OS, overall survival; R, rituximab; R-CHOP, rituximab–cyclophosphamide, doxorubicin hydrochloride, vincristine, and prednisone; R-DHAOx, rituximab–dexamethasone, high-dose cytarabine, and oxaliplatin; R-DHAP, rituximab–dexamethasone, high-dose cytarabine, and cisplatin; *TP53*m, TP53 mutation.

**Table 3:** Case 2: Key clinical features and treatment options under consideration for a patient with standard risk MCL—Scenario A.

**Key Clinical Features:** A 75-year-old male Diagnosis: Previously untreated stage IV standard-risk MCL, now with symptomatic splenomegaly ECOG PS: 1 MIPI: 6.1 (intermediate risk) ^a^ Morphology: Classic Ki-67: 40% *TP53*m status: Wild type Past medical history: Hypertension, gastroesophageal reflux disease			
**SCENARIO A: Treatment Options Under Consideration**			
**BR (reserve cBTKi for relapse)** **(StiL-1 and BRIGHT trials;** **SHINE/ECHO trial control arms)**		**BR + cBTKi Combination** **(SHINE and ECHO trials)**	
**Key Advantages**	**Key Disadvantages**	**Key Advantages**	**Key Disadvantages**
Favorable long-term toxicity profile and possibility for disease control in some patients without concomitant cBTKi therapy Fixed-duration therapy with potential for treatment break and improved toxicity and costs Ensures availability of cBTKi or I + V in 2L	Shorter PFS Patient may not be eligible for eventual 2L cBTKi therapy due to disease biology or comorbidities	Superior PFS vs. BR alone Appropriate for patients without major comorbidities Opportunity to access a second-generation cBTKi (ECHO)	Potential toxicity, especially with ibrutinib Lack of OS benefit Indefinite cBTKi therapy increases safety risks, HCRU, costs, and treatment resistance Limited treatment options upon disease progression

^a^ Cut-offs based on Hoster et al. (2008) [35]. 2L, second-line; BR, bendamustine + rituximab; cBTKi, covalent Bruton’s tyrosine kinase inhibitor; ECOG, Eastern Cooperative Oncology Group performance status; HCRU, healthcare resource utilization; I, ibrutinib; MCL, mantle cell lymphoma; MIPI, MCL International Prognostic Index; OS, overall survival; PFS, progression-free survival; *TP53*m, *TP53* mutation; V, venetoclax.

## Data Availability

No new data were created or analyzed in this study. Data sharing is not applicable to this article.

## Supplementary Materials

The following supporting information can be downloaded at: https://www.mdpi.com/article/10.3390/curroncol33070426/s1, Table S1: Additional phase II clinical trials evaluating 1L cBTKi combinations in MCL.

## Abbreviations

The following abbreviations are used in this manuscript:

XL	X line of therapy
A	Acalabrutinib
AE	Adverse event
AlloSCT	Allogeneic stem cell transplantation
ASCT	Autologous stem cell transplantation
BCL-2(i)	B-cell lymphoma 2 (inhibitor)
BR	Bendamustine + rituximab
CAR	Chimeric antigen receptor
cBTKi	Covalent Bruton’s tyrosine kinase inhibitor
CI	Confidence interval
CIT	Chemoimmunotherapy
CR(R)	Complete response (rate)
CV	Cardiovascular
ECOG PS	Eastern Cooperative Oncology Group performance status
FFS	Failure-free survival
GVHD	Graft-versus-host disease
HCRU	Healthcare resource utilization
HR	Hazard ratio
HRQoL	Health-related quality of life
IHC	Immunohistochemistry
I	Ibrutinib
I + V	Ibrutinib + venetoclax
MCL	Mantle cell lymphoma
MIPI	Mantle cell lymphoma international prognostic index
Mo	Month(s)
MRD	Minimal residual disease
NGS	Next-generation sequencing
N/A	Not available
NR	Not reached
NRM	Non-relapse mortality
ORR	Overall response rate
OS	Overall survival
PBO	Placebo
PD	Progressive disease
PFS	Progression-free survival
R-CHOP	Rituximab–cyclophosphamide, doxorubicin hydrochloride, vincristine, prednisone
R-DHAOx	Rituximab–dexamethasone, high-dose cytarabine, oxaliplatin
R-DHAP	Rituximab–dexamethasone, high-dose cytarabine, cisplatin
R/R	Relapsed/refractory
SDM	Shared decision-making
*TP53*m	*TP53* mutation
uMRD	Undetectable minimal residual disease
V	Venetoclax
yr(s)	Year(s)
Z	Zanubrutinib
