# Supplementary material for: First-Line Bruton’s Tyrosine Kinase Inhibitor-Based Regimens for Mantle Cell Lymphoma
Source: Curr Oncol. 2026 Jul 17;33(7):426. doi: 10.3390/curroncol33070426 (PMC13409616; doi:10.3390/curroncol33070426)
Supplement: Supplementary file 1 [file curroncol-33-00426-s001.zip › curroncol-4368728-supplementary.pdf]

Supplementary Data

First-line Bruton's Tyrosine Kinase Inhibitor-based Regimens for Mantle Cell Lymphoma

Robert Puckrin [1*], Diego Villa [2,3], Isabelle Fleury [4], Jean-François Larouche [4] and John Kuruvilla[5]

1 Arthur J.E. Child Comprehensive Cancer Centre, University of Calgary, Calgary, AB, T2N 5G2,Canada
2 Centre for Lymphoid Cancer, BC Cancer, Vancouver, BC, V5Z 1L3, Canada
3 Division of Medical Oncology, Department of Medicine, University of British Columbia, Vancouver, BC, V5Z 4E6, Canada
4 Centre Hospitalier Universitaire de Québec, Université Laval, Québec City, QC, G1J 5B3, Canada

5 Division of Medical Oncology and Hematology, Princess Margaret Cancer Centre, Toronto, ON, M5G 2M9, Canada;

* Correspondence: robert.puckrin@albertahealthservices.ca

Table S1. Additional phase II clinical trials evaluating 1L cBTKi combinations in MCL

Study Name/Group<br>NCT Number<br>Author, Year	Intervention	Population<br>*n*<br>Median Age	Transplant Eligibility	Risk Characteristics[a]				Median Follow-up (mo)	ORR	CRR	PFS	OS
				MIPI High	Ki-67 ≥30%	*TP53*	Blastoid/ Pleomorphic Variants					
**Chemotherapy Combinations**												
NHL33 'Wamm'<br>ACTRN 12619000990123<br>Hawkes et al 2023 (Abs) [1]	AR (2-cycle window), R-DHAOx ± ASCT, then AR maint	Fit,<br>18-70 yrs<br>*n* = 44<br>59 yrs	Eligible	N/A	>30%:<br>66%	N/A	9%	22	Post-induction:<br>93%<br><br>Post A+R+ R-DHAOx:<br>95%	Post-induction:<br>57%<br><br>Post A+R+ R-DHAOx:<br>88%	N/A	2-yr:<br>89%
WINDOW-1<br>NCT02427620<br>Wang et al 2022 [2]	Part A: IR;<br>Part B: if CR, R-HCVAD alternating with MTX-ARA-C	≤65 yrs<br>*n* = 131<br>56 yrs	Eligible	s-MIPI:<br>8%<br>MIPI-b:<br>36%	49%<br>(58/117)	*TP53*m:<br>32%<br>(11/34)	11%	42	Part A: 98%<br>(best)<br>Part A+B:<br>90%	Part A: 87%<br>Part A+B:<br>89%	3-yr:<br>0.79	3-yr:<br>0.95
WINDOW-2<br>NCT03710772<br>Wang et al 2023 (Abs) [3]	Part 1: IRV<br>Part 2:<br>R-HCVAD/ MTX-ARA-C consolidation (risk-stratified) and IRV maint	≤65 yrs<br>*n* = 50<br>58 yrs	Eligible	N/A	N/A	N/A	N/A	41	Part 1:<br>100% (best)	Part 1:<br>100%	3-yr:<br>85%	3-yr:<br>86%
CHESS<br>NCT04624958<br>Cai et al 2024 (Abs) [4]	ZR,<br>R-DHAOx (short course), then Z maint	Not specified<br>*n* = 42<br>57 yrs	Not specified	s-MIPI:<br>7.1%	N/A	N/A	Blastoid, small cell, + pleomorphic<br>19%	11.6	N/A	91.9%<br>(best post-Z+R)	1-yr:<br>90.1%	1-yr:<br>96.7%
ECOG-ACRIN EA4181<br>NCT04115631<br>Wagner-Johnston et al 2024 (Abs) [5]	BR/CR (control) vs. BR/CR+A vs. BR+A	≤70 yrs<br>*n* = 359<br>61 yrs	Eligible	39%	N/A	N/A	N/A	27.9	BR/CR:<br>94%<br>BR/CR+A:<br>99%<br>BR+A:<br>94%	N/A	12-mo:<br>BR/CR:<br>86%<br>BR/CR+A:<br>89%<br>BR+A:<br>87%	12-mo:<br>BR/CR:<br>94%<br>BR/CR+A<br>98%<br>BR+A:<br>95%

Study Name/Group<br>NCT Number<br>Author, Year	Intervention	Population<br>*n*<br>Median Age	Transplant Eligibility	Risk Characteristics[a]				Median Follow-up (mo)	ORR	CRR	PFS	OS
				MIPI High	Ki-67 ≥30%	*TP53*	Blastoid/ Pleomorphic Variants					
**Chemotherapy-free Combinations**												
IMCL-2015/GELTAMO<br>NCT02682641<br>Giné et al 2022 [6]	IR	Indolent MCL<br>≥18 yrs<br>*n* = 50<br>65 yrs	Not specified	38%	5%	*TP53*m:<br>15%<br>(6/41)	0%	36	C12: 84%	C12: 80%	3-yr:<br>93%	3-yr:<br>92%
MD Anderson<br>NCT05214183<br>Jain et al 2023 (Abs) [7]	AR	Elderly<br>(≥65 yrs)<br>*n* = 50<br>69 yrs	Not specified	s-MIPI:<br>22%	31%	*TP53*m:<br>28%<br>(12/43)	8%	17	94%<br>(best)	90%<br>(best)	2-yr:<br>92%	2-yr:<br>96%
NCT03863184<br>Ruan et al 2024<br>(Abs) [8]	ALR	Unselected<br>*n* = 24<br>64 yrs	Not specified	29%	>30%:<br>36%	*TP53*m:<br>29%	N/A	41	100%	83%	3-yr:<br>88%	3-yr:<br>95%
ChiCTR2300071433<br>Zhu et al 2024<br>(Abs) [9]	ZO,<br>then Z maint	Elderly<br>(≥65 yrs)<br>*n* = 19<br>72 yrs	Not specified	39%	53%	*TP53*m:<br>27%<br>(4/15)	Excluded	7	100%[a]	88.9%[a]	N/A	N/A
OAsIs<br>NCT02558816<br>Le Gouill et al 2021 [10]<br>Tessoulin et al 2024 [11]	IOV	>18 yrs<br>*n* = 15<br>(Cohort C)<br>65 yrs	Not specified	27%<br>(b-MIPI:<br>55%)	N/A	*TP53*m:<br>13%<br>(n=2)<br>*del17p*:<br>40%<br>(n = 6)	7%<br>(n=1)	61	93%	87%	5-yr:<br>80%	5-yr:<br>93%
OAsIs II<br>NCT04802590<br>Le Gouill et al 2024/2025 (Abs)<br>[12, 13]	Arm A:<br>I + anti-CD20<br>Arm B:<br>I + anti-CD20 + V	18-80 yrs<br>*n* = 102<br>Arm A:<br>67 yrs<br>Arm B:<br>63 yrs	Not specified	Arm A:<br>39%<br>Arm B:<br>31%	>30% (n):<br>Arm A:<br>9<br>Arm B: 16	N/A	Blastoid (n):<br>Arm A: 1<br>Arm B: 3	N/A	N/A	N/A	2-yr:<br>87.9%<br>(Arms A+B)	2-yr:<br>91.9%<br>(Arms A+B)

Study Name/Group NCT Number Author, Year	Intervention	Population n Median Age	Transplant Eligibility	Risk Characteristics[a]				Median Follow-up (mo)	ORR	CRR	PFS	OS
				MIPI High	Ki-67 ≥30%	*TP53*	Blastoid/ Pleomorphic Variants					
Kumar et al 2025 (Abs) [14]	ZOV Treatment D/C based on uMRD6 after 24 cycles	≥65 yrs or comorbidities precluding ASCT *n* = 50 72 yrs	Ineligible	70%	51%	*TP53*m: 28%<br>*del17p*: 20%	Blastoid: n=6	25	98% (best)	94%	2-yr: 86%	2-yr: 92%
ZR Study NCT05504603 Qu et al 2025 [15]	ZR, then Z maint. or ASCT	Not specified *n* = 39 61 yrs	Mixed	>50% intermediate-risk IPI	N/A	*TP53*m: 15.4% (6/26)	N/A	22.6	C3: 94.9%	C3: 84.6% C5: 100% (36/36)	2-yr: 94.9%	2-yr: 100%

Information presented from most recent source(s)/data cuts available at the time of publication. Cross-trial comparisons should be conducted with caution given differences in study designs, patient populations, interventions, endpoint definitions, and methods of analysis.

[a] Denominator represents 9 patients who had completed induction therapy at the time of the analysis.

1L, first line; A, acalabrutinib; Abs, abstract only; AE, adverse events; ASCT, autologous stem cell transplantation; B, bendamustine; C, cytarabine; CX, cycle number, cBTKi, covalent Bruton's tyrosine kinase inhibitor; CI, confidence interval; CR, cytarabine-rituximab; CRR, complete response rate; CTX, chemotherapy; D/C, discontinuation; HR, hazard ratio; I, ibrutinib; IPI, International Prognostic Index; L, lenalidomide; maint., maintenance; (s/b/c)-MIPI, (simplified, biologic, or combined) Mantle Cell Lymphoma International Prognostic Index; MTX-ARA-C, methotrexate + cytarabine; N/A, not available; NR, not reached; O, obinutuzumab; OR, objective response; ORR, objective/overall response rate: OS, overall survival; PFS, progression-free survival; R, rituximab; R-DHAOx, rituximab–dexamethasone, high-dose cytarabine, and oxaliplatin; R-HCVAD, rituximab–hyperfractionated, cyclophosphamide, vincristine, doxorubicin hydrochloride, and dexamethasone; *TP53*m, *TP53* mutation; uMRD(6), undetectable minimal residual disease (at 10^{-6}); V, venetoclax; yr(s), year(s); Z, zanubrutinib.

References

1. Hawkes, E.A.; Lee, S.T.; Churilov, L.; Armytage, T.; Hodges, G.; Cheah, C.Y.; Wight, J.; Wong Doo, N.; Ku, M.; Opat, S.S.; Cochrane, T.; Hapgood, G.; Lee, H.-P.; Agarwal, R.; Lin, W.; Koshy, M.; Walia, M.; Olenko, L.; Yeh, P.S.-H.; Devitt, B.; Barraclough, A.A. A window study of acalabrutinib & rituximab, followed by chemotherapy & autograft (ASCT) in fit patients with treatment naïve mantle cell lymphoma (MCL): first report of the investigator-initiated Australasian Leukaemia & Lymphoma Group NHL33 'Wamm' trial. *Blood.* **2023**, *142*, 735.
2. Wang, M.L.; Jain, P.; Zhao, S.; Lee, H.J.; Nastoupil, L.; Fayad, L.; Ok, C.Y.; Kanagal-Shamanna, R.; Hill, H.A.; Yao, Y.; Hagemeister, F.B.; Westin, J.R.; Fowler, N.; Samaniego, F.; Steiner, R.; Nair, R.; Iyer, S.P.; Navsaria, L.; Badillo, M.; Feng, L.; Xuelin, H.; Nogueras Gonzalez, G.M.; Xu, G.; Wagner-Bartak, N.; Thirumurthi, S.; Santos, D.; Tang, G.; Lin, P.; Wang, S.A.; Jorgensen, J.; Yin, C.C.; Li, S.; Patel, K.P.; Vega, F.; Medeiros, L.J.; Flowers, C.R.; Wang, L. Ibrutinib-rituximab followed by R-HCVAD as frontline treatment for young patients (≤65 years) with mantle cell lymphoma (WINDOW-1): a single-arm, phase 2 trial. *Lancet Oncol.* **2022**, *23*, 406-415.
3. Wang, M.L.; Lee, H.J.; Fetooh, A.; Nair, R.; Ok, C.Y.; Hill, H.A.; Iyer, S.P.; Steiner, R.E.; Westin, J.; Nastoupil, L.; Fowler, N.; Kanagal-Shamanna, R.; Jelloul, F.Z.; Castillo, L.E.M.; Liu, Y.; Li, Y.; Vargas, J.; Feng, L.; Badillo, M.; Spike, T.; Thirumurthi, S.; Santos, D.; Xu, G.; Deswal, A.; Tang, G.; Patel, K.P.; Vega, F.; Medeiros, L.J.; Flowers, C.; Jain, P. Ibrutinib-rituximab and venetoclax (IRV) followed by risk-stratificed R-hyper-CVAD/MTX in young patients with untreated mantle cell lymphoma--phase-II WINDOW-2 trial. *Hematologl Oncol.* **2023**, *41*, 152-153.
4. Cai, Q.; Xia, Y.; Huang, H.; Li, Z.; Huang, H.; Li, G.; Zhang, Y.; Cao, Y.; Gao, Y.; Liu, P.; Fang, X.; Mie, M.; Tian, X.; Sun, X.; Pan, X. Frontline treatment with zanubrutinib plus rituximab (ZR) followed by short course R-DHAOx in patients with mantle cell lymphoma (MCL): results of the phase II CHESS clinical trial. *J. Clin. Oncol.* **2024**, *42*, 7062.
5. Wagner-Johnston, N.; Jegede, O.; Spurgeon, S.E.; Maddocks, K.J.; Yang, D.T.; Romanoff, J.; Paludo, J.; Caimi, P.F.; Bartlett, N.L.; Reagan, P.M.; Sawalha, Y.; Hoffmann, M.; Hu, B.; Aziz, A.R.; Marques, H.; Kosakoglu Shields, L.; Leonard, J.P.; Friedberg, J.W.; Kahl, B.S. Addition or substitution of acalabrutinib in intensive frontline chemoimmunotherapy for patients ≤70 years old with mantle cell lymphoma: outcomes of the 3-arm randomized phase II Intergroup trial ECOG-ACRIN EA4181. *Blood.* **2024**, *144*, 236.
6. Giné, E.; de la Cruz, F.; Jiménez Ubieto, A.; López Jimenez, J.; Martín García-Sancho, A.; Terol, M.J.; González Barca, E.; Casanova, M.; de la Fuente, A.; Marín-Niebla, A.; Muntañola, A.; González-López, T.J.; Aymerich, M.; Setoain, X.; Cortés-Romera, M.; Rotger, A.; Rodríguez, S.; Medina Herrera, A.; García Sanz, R.; Nadeu, F.; Beà, S.; Campo, E.; López-Guillermo, A. Ibrutinib in combination with rituximab for indolent clinical forms of mantle cell lymphoma (IMCL-2015): a multicenter, open-label, single-arm, phase II trial. *J. Clin. Oncol.* **2022**, *40*, 1196-1205.
7. Jain, P.; Young Ok, C.; Nastoupil, L.J.; Westin, J.; Hill, H.A.; Nair, R.; Iyer, S.P.; Fetooh, A.; Lee, H.J.; Ahmed, S.; Kanagal-Shamanna, R.; Jelloul, F.Z.; Malpica Castillo, L.E.; Liu, Y.; Li, Y.; Vargas, J.; Feng, L.; Badillo, M.; Thirumurthi, S.; Xu, G.; Deswal, A.; Iliescu, C.; Quang Nguyen, V.; Tang, G.; Patel, K.P.; Vega, F.; Medeiros, L.J.; Wang, M.L.; Flowers, C.R. Acalabrutinib with rituximab as first-line therapy for older patients with mantle cell lymphoma - a phase II clinical trial. *Blood.* **2023**, *142*, 3036.
8. Ruan, J.; Bond, D.A.; Shah, B.D.; Allan, J.N.; Rutherford, S.C.; Gribbin, C.; Chen, Z.; Hobbie, B.; Harbhajan, M.; Sahni, T.; Tam, W.; Bhinder, B.; Sigouros, M.; Inghirami, G.; Chen-Kiang, S.; Elemento, O.; Maddocks, K.J.; Leonard, J.P.; Martin, P. MRD-driven time-limited therapy of acalabrutinib and lenalidomide plus rituximab (ALR) or obinutuzumab (ALO) in patients with treatment-naïve mantle cell lymphoma: phase 2 trial outcomes with MRD and cfDNA analyses. *Blood.* **2024**, *144*, 746.

9. Zhu, Y.; Lv, Y.; Ye, X.; Wei, J.; Sheng, L.; Lin, Y.; Zhang, X.; Zhang, X.; Tong, H.; Jin, J.; Yu, W.-J. The efficacy and safety of obinutuzumab with zanubrutinib as first-Line treatment in older patients with mantle cell lymphoma. *Blood.* **2024**, *144*, 1630.
10. Le Gouill, S.; Morschhauser, F.; Chiron, D.; Bouabdallah, K.; Cartron, G.; Casasnovas, O.; Bodet-Milin, C.; Ragot, S.; Bossard, C.; Nadal, N.; Herbaux, C.; Tessoulin, B.; Tchernonog, E.; Rossi, C.; McCulloch, R.; Gastinne, T.; Callanan, M.B.; Rule, S. Ibrutinib, obinutuzumab, and venetoclax in relapsed and untreated patients with mantle cell lymphoma: a phase 1/2 trial. *Blood.* **2021**, *137*, 877-887.
11. Tessoulin, B.; Morschhauser, F.; Chiron, D.; Bouabdallah, K.; Cartron, G.; Rossi, C.; Milin, C.; Fouillet, L.; Herbaux, C.; Tchernonog, E.; McCulloch, R.; Gastinne, T.; Callanan, M.B.; Le Gouill, S. 5-y follow-up of combination of ibrutinib, obinutuzumab and venetoclax for patients with newly diagnosed mantle cell lymphoma, the OAsIs trial. *Blood.* **2024**, *144*, 1657.
12. Le Gouill, S.; de Wilde, V.; Eyre, T.A.; Callanan, M.; Damaj, G.L.; Claudel, A.; Adrien, C.; Houot, R.; Le Calloch, R.; Cacheux, V.; Andre, M.; Morschhauser, F.; Burroni, B.; Lewis, D.J.; Tessoulin, B.; Touzart, A. Ibrutinib, venetoclax plus CD20 monoclonal Ab: initial results of OASIS II, a prospective randomized phase 2 trial in previously untreated mantle cell lymphoma patients. *J. Clin. Oncol.* **2025**, *43*, 7044.
13. Le Gouill, S.; de Wilde, V.; Eyre, T.A.; Callanan, M.; Damaj, G.L.; Claudel, A.; Adrien, C.; Houot, R.; Le Calloch, R.; Cacheux, V.; Andre, M.; Morschhauser, F.; Burroni, B.; Lewis, D.J.; Tessoulin, B.; Touzart, A. Ibrutinib, venetoclax plus CD20 monoclonal Ab provides very high MRD negativity in previously untreated MCL patients, initial results of OASIS II, a randomized phase 2 trial. Abstract 745. 66th ASH Annual Meeting & Exposition. Available online: https://ash.confex.com/ash/2024/webprogram/Paper194357.html (accessed on 26 June 2026).
14. Kumar, A.; Soumerai, J.; Karmali, R.; Abramson, J.; Barnes, J.; Beatty, B.; et al. Preliminary safety and efficacy of BOven (zanubrutinib, obinutuzumab, and venetoclax) as frontline therapy for older patients with mantle cell lymphoma. Abstract #888 (presentation). 67th ASH Annual Meeting and Exposition. December 6-9, 2025. Orlando, FL
15. Qu, C.-J.; Ping, N.-N.; Zou, R.; He, J.-J.; Zhu, Q.; Zhang, X.; Xia, F.; Kong, D.; Yu, L.; Wang, Y.; Dong, X.; Wu, D.; Jin, Z. ZR study: a prospective phase II clinical trial evaluting zanubrutinib-rituximab induction therapy with or without autologous stem cell transplantation in treatment-naive mantle cell lymphoma. PS1871. EHA 2025. June 12-15, 2025. Milan, Italy.
